# Parsing a Cognitive Task: A Characterization of the Mind's Bottleneck

**DOI:** 10.1371/journal.pbio.0030037

**Published:** 2005-02-08

**Authors:** Mariano Sigman, Stanislas Dehaene

**Affiliations:** **1**Unité INSERM 562, Cognitive NeuroimagingService Hospitalier Frédéric Joliot, CEA/DRM/DSV, Orsay cedexFrance; University of OregonUnited States of America

## Abstract

Parsing a mental operation into components, characterizing the parallel or serial nature of this flow, and understanding what each process ultimately contributes to response time are fundamental questions in cognitive neuroscience. Here we show how a simple theoretical model leads to an extended set of predictions concerning the distribution of response time and its alteration by simultaneous performance of another task. The model provides a synthesis of psychological refractory period and random-walk models of response time. It merely assumes that a task consists of three consecutive stages—perception, decision based on noisy integration of evidence, and response—and that the perceptual and motor stages can operate simultaneously with stages of another task, while the central decision process constitutes a bottleneck. We designed a number-comparison task that provided a thorough test of the model by allowing independent variations in number notation, numerical distance, response complexity, and temporal asynchrony relative to an interfering probe task of tone discrimination. The results revealed a parsing of the comparison task in which each variable affects only one stage. Numerical distance affects the integration process, which is the only step that cannot proceed in parallel and has a major contribution to response time variability. The other stages, mapping the numeral to an internal quantity and executing the motor response, can be carried out in parallel with another task. Changing the duration of these processes has no significant effect on the variance.

## Abstract 2

The inability to operate two cognitive processes simultaneously - a mental bottleneck - can be explained by a model in which evidence is accumulated stochastically to reach a decision

## Introduction

Even the most simple behaviour involves a chain of computations, which link perception, decision making, and action [1,2,3]. Measurements of response times (RTs) have been used as a major source of information on the organization of these stages [4,5], and more recently these analyses have been combined with neuroimaging data to identify separate processing modules [6,7]. This seemingly simple measure of time to completion of a cognitive operation has several intriguing properties. One of them is its noisy character. Even in very simple tasks, RTs typically vary over a broad range of several hundred milliseconds. Another property is that RT can slow down considerably under some circumstances in which the subject is distracted by another competing stimulus or task. This suggests the existence of at least some stages that act as a bottleneck and can only operate serially, one at a time. Here we set out to relate response variability and the serial versus parallel architecture of processing stages. Do all stages of processing contribute uniformly to this variance? Or are some stages particularly variable in their computation time? And does variability relate in a systematic manner to their parallel or serial nature?

When two tasks are presented simultaneously (or sequentially at a short interval), a delay in the execution of the second task has been systematically observed [8,9,10,11]. This interference effect is referred to as the psychological refractory period (PRP) and has been explained by a model that involves three stages of processing: a perceptual component (P component), a central component (C component), and a motor component (M component), in which only the C component establishes a bottleneck [5,9,12,13,14,15]. PRP experiments have associated the C component to “response selection”, the mapping between sensory information and motor action [16].

A separate line of psychological research has investigated how the decision to respond is achieved. The decision-making process has been modelled as a noisy integrator that accumulates evidence provided by the sensory system [17,18,19,20,21,22,23,24,25]. Although many variants have been proposed, the basic idea is that perceptual evidence is stochastically accumulated in time. Decision thus results from a random walk of an internal abstract variable. Indeed, in many circumstances, such a decision mechanism can be optimal in the sense that it maximizes the overall likelihood of a correct classification of the stimuli [26,27]. In the simplest scheme, all the variance in RT is attributed to this integration process. Thus, the integration model establishes a possible parsing of a task into components: a fixed component to transform the sensory information to an abstract variable, the accumulation of evidence itself (the only variable process), and the execution of the response.

**Figure 1:**
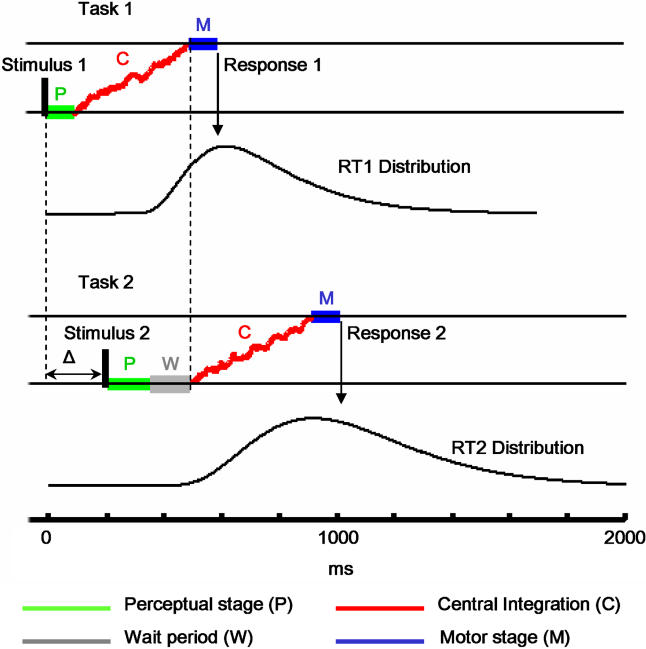
The Model: The Process of Accumulation of Evidence Constitutes the Mind's Bottleneck Each task involves a sequence of three stages of processing. The perceptual and motor stages are fixed and can be carried out in parallel with stages of another task, while the central stage consists of a noisy integration (a random walk) until a decision threshold is reached. The central stage of task 2 cannot start until the central stage of task 1 is finished. Thus, this establishes a bottleneck and characterizes a serial process. The distribution of RTs for the second task is wider than that for the first task, because it combines the intrinsic variance of task 2 (the time to reach threshold) and the variance in onset of the central stage of task 2, which is set by the ending of the central stage of task 1.

In the present work, we propose a single assumption that unifies those two lines of research. We postulate that only the integration process establishes a serial bottleneck, while all other stages can proceed in parallel with stages of another task (Figure 1). This model should thus explain both the dual-task interference experiments and the detailed analysis of RT distributions. While extremely simple, the model makes powerful mathematical predictions in experiments in which the order of the presentation of the two tasks and their relative offset in presentation are varied. Moreover, it also makes specific predictions in experiments in which the complexity of one of the tasks is changed. Depending on whether the locus of the change is in the P, C, or M components, the shape of RTs as a function of the delay between stimuli acquires a very different shape.

We designed a behavioural task to test the validity of the model. This number-comparison task involves deciding whether a digit presented on the screen is larger or smaller than 45. Different manipulations of the task render it more difficult, presumably, at different stages of processing. The different task manipulations include notation (whether the number was presented in Arabic digits or in spelled words), distance (the numerical distance between the presented number and 45), and response complexity (whether subjects were asked to tap once or twice to indicate their choice). Previous studies have shown that all of these manipulations change the difficulty of the task: RTs increase when numerical distance decreases and when numbers are presented in spelled words [28,29]. These effects have been shown to be additive and to involve distinct brain regions and components of the event-related potentials [29,30]. Thus, it is likely that they affect different components of processing, making this task a good candidate to explore the validity of the model.

## Results

Subjects were asked to perform a dual task. One of the two tasks was presented visually and involved a number comparison: subjects decided whether a digit presented on the screen was larger or smaller than 45. Hereafter we will refer to it as “number task”. The other was a tone-discrimination task that involved deciding whether the frequency of a single pure tone that was presented for 150 ms was high (880 Hz) or low (440 Hz) (subjects heard both tones repeatedly before the beginning of the experience). Hereafter we will refer to it as “tone task”. Two different populations of subjects performed the task in the two possible orders, tone task followed by number task or vice versa.

The number task was our main task of study, and was manipulated using three different factors: notation (whether the number was presented in Arabic digits or in spelled words), distance (the numerical distance between the presented number and 45), and response complexity (whether subjects were asked to tap once or twice to indicate their choice). The tone task was never varied throughout the experiment. The rationale underlying this experimental design is that the tone task is used as a probe to study, through interference experiments, the different stages of processing of the number task. This asymmetry between the two tasks, which might be helpful to keep in mind, was of course not stated to the subjects, who were just asked to attend equally to both tasks.

The results section is organized as follows. We first report an analysis of basic measures of central tendency and dispersion. We then address how different manipulations (within the number task or through the interference with the tone task) change the mean RTs and their dispersion. These types of tests allow us to test the additivity of the effects of each factor and, through the interference analysis, whether they affect the perceptual, the central, or the motor stage. A second level of analysis involves a more detailed characterization of the shapes of the distributions of RTs. Fitting the distributions allows us to evaluate the accumulation model of response decision and to relate its components to those identified by their patterns of interference in the first-level analysis.

For clarity and as a reference throughout the paper, all the definitions, components of the models, and experimental manipulations are summarized in Table 1.

**Table 1:**
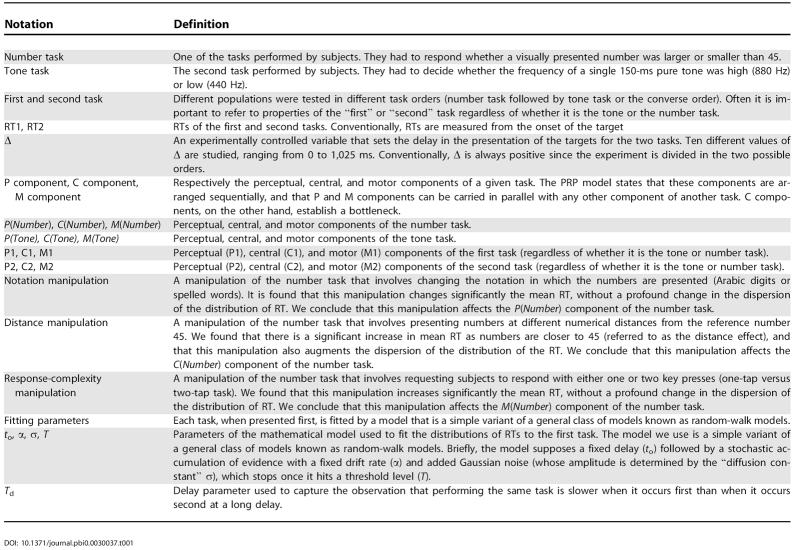
Definitions of Notations

DOI: 10.1371/journal.pbi0.0030037.t001

### Analysis of Mean RTs and Interquartile Range

#### Effect of the different manipulations of the number task

The first analysis involved studying the effects of the different manipulations of notation, distance, and response complexity on mean RTs and response dispersion on the number task when it came first. Our model predicted that manipulations that affect separate stages should have additive effects on mean RTs, and that only manipulations that affect the central stage should significantly increase response dispersion.

For this analysis (and throughout the paper unless otherwise specified) distance, which is the absolute value of the difference between the presented number and 45, was binned in two groups, close (≤12) and far (>12). Central tendency was measured by estimating the mean RT after trimming for outliers, by discarding responses slower than 1,200 ms. Response dispersion was measured by estimating the interquartile range, i.e., the difference between the 75th percentile and 25th percentile of the distribution of RTs. (Identical results were obtained when using other measures, e.g., median and standard deviation of RTs. Note that, in general, serial stage models predict that factors affecting distinct stages should have additive effects on mean RTs, but not necessarily on median RTs. Our model, however, supposes that factors affecting P and M components do not add to response dispersion, but merely add a constant factor to RTs. Under this hypothesis, factors affecting selectively P, C, and M components should also have additive effects on median RTs. In fact, the effects of perceptual and motor factors should be quantitatively the same on mean and median RTs.)

**Figure 2:**
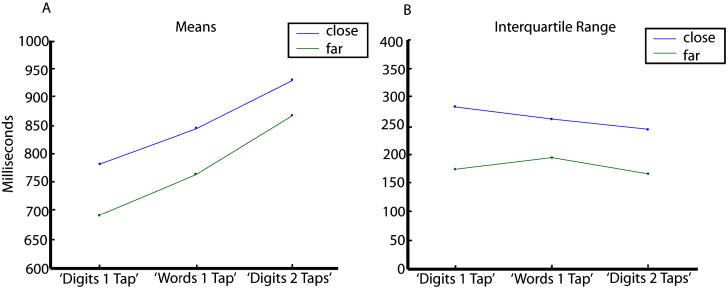
Effects of the Different Manipulations on the Mean and Dispersion of RT (A) Changes in the mean RT of the numeric task when it comes first in the different experimental manipulations. Changing the notation or the response complexity makes mean RT slower, and within each condition, responses are slower for close than for far distances. The difference between far and close conditions is independent of the experimental manipulation, indicating an additive effect that is tested in the ANOVAs (see Table 2). (B) A different pattern is observed for the interquartile range, which provides a measure of dispersion. While distance manipulation results in a major change of the interquartile range, there is not a major effect of notation or response complexity.

As we expected from several prior experiments [29,30], performing the task for spelled words required more time than for Arabic digits (Figure 2A). We also observed a significant distance effect: RT for close numbers was longer than for far numbers. In addition, these effects were additive, as revealed by ANOVAs with subjects as a random factor and notation and distance as within-subject factors (Table 2, effects of notation; Figure 2A). Similarly, response complexity increased subjects' mean RT, and this effect was additive with the distance manipulation (Table 2, effects of response complexity; Figure 2A).

**Table 2:**
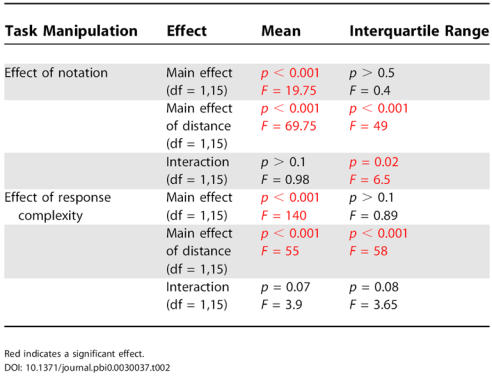
Results of the Different Manipulations of the Number Task on the Mean and Interquartile Range

Red indicates a significant effect

DOI: 10.1371/journal.pbi0.0030037.t002

Interestingly the effects of the different manipulations on response dispersion did not follow the effects on the mean, indicating that some factors slowed RT but did not significantly increase their dispersion. The distance manipulation resulted in a significant increase of the interquartile range typical of stochastic process, where the dispersion increases with the mean. In contrast, notation and response complexity, while causing an important change in the mean, did not result in a significant increase of the interquartile range (Table 2; Figure 2B).

To fully address whether the number-comparison task involves three separate stages with each experimental factor (distance, notation, and response complexity), a complete “additive factors” experimental design is needed, in which the different factors are crossed and thus all the interactions can be tested. However, such a factorial design, if tested in the double-task experiment, would involve an exceedingly large number of conditions, which would be very difficult to test on a subject by subject basis within a single session. Instead, we ran it as an independent experiment, in which a new group of subjects was asked to perform only the number task.

The results of this new experiment, summarized in Table 3, confirmed and extended our previous findings. (1) All factors (distance, notation, and response complexity) had a significant main effect on mean RT. (2) All interactions between factors were nonsignificant. In particular, the new experiment allowed us to test two interactions that could not be addressed previously (notation by response complexity and triple interaction), which were also nonsignificant. This additive-factors analysis is thus fully compatible with our hypothesis that the number task involves three successive stages, each selectively influenced by one of the three factors. (3) As expected, those findings held for both analyses of the mean and median RTs, both of which are reported in Table 3. (4) As shown previously, the interquartile range and standard deviation were only affected by the distance manipulation, but did not change significantly with the response complexity and notation manipulations. The size of the confidence intervals was similar in all conditions (Table 3), suggesting that this was not just a matter of statistical power. For instance, response complexity had a major impact on mean RT (249 ± 20 ms), but no effect on its standard deviation (1.4 ± 8.9 ms), while distance had a more modest impact on mean RT (41.2 ± 5.8 ms) and a comparable effect on standard deviation (19.2 ± 6.5 ms). While this result does not necessarily imply that the variance associated with notation- and response-dependent processes is strictly zero, it suggests that those processes have a substantially lower contribution to the variance than the distance-dependent stage.

**Table 3:**
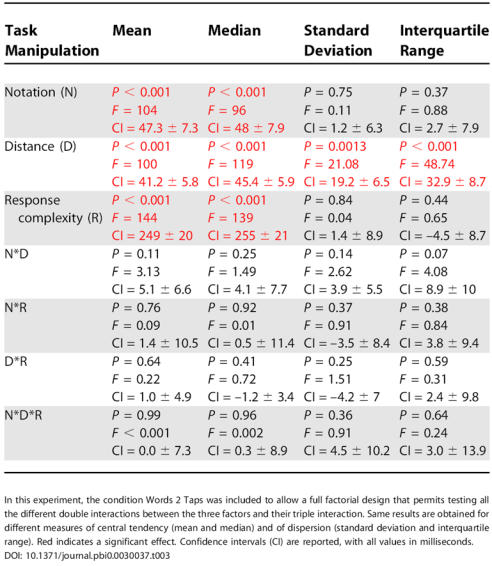
Results of the Different Manipulations of the Number Task (in a Single-Task Experiment) on Different Variables

In this experiment, the condition Words 2 Taps was included to allow a full factorial design that permits testing all the different double interactions between the three factors and their triple interaction. Same results are obtained for different measures of central tendency (mean and median) and of dispersion (standard deviation and interquartile range). Red indicates a significant effect. Confidence intervals (CI) are reported, with all values in milliseconds

DOI: 10.1371/journal.pbi0.0030037.t003

Taken together with the assumption of our model that only the central stage contributes to response variance, our observations suggest that the numerical distance factor affects the C decision component, while notation and response complexity affect noncentral P or M components.

#### Interference by the tone task

**Figure 3:**
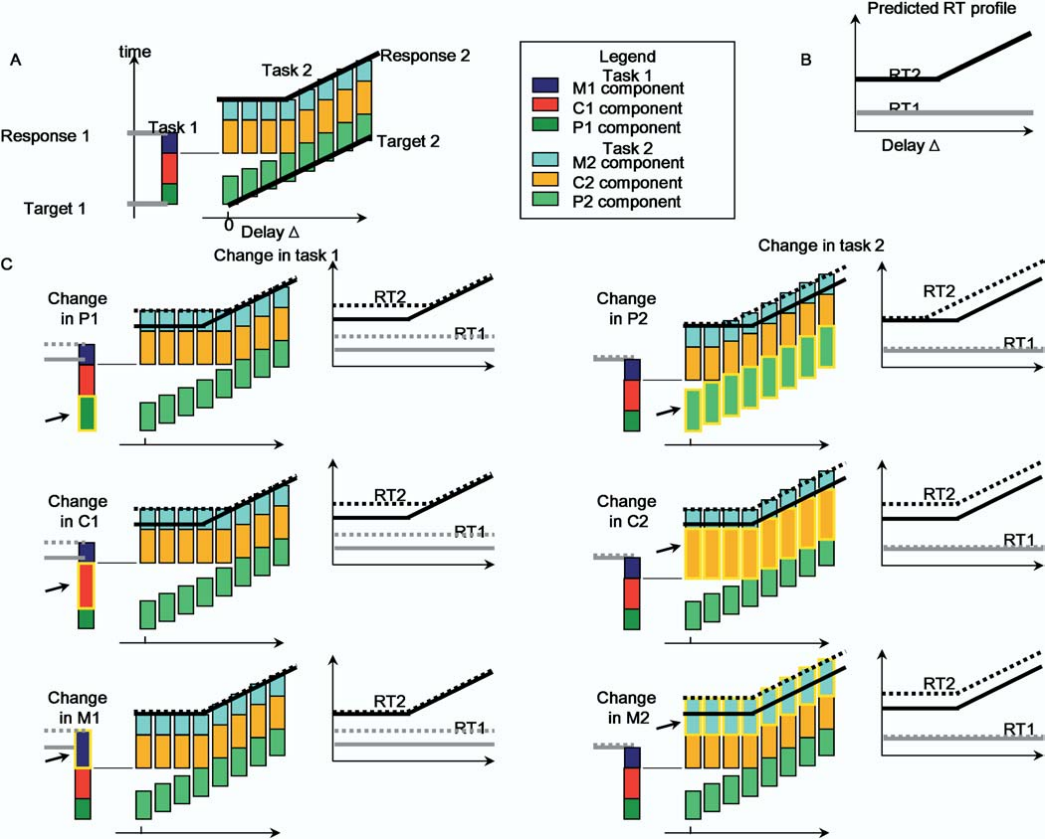
Description of the Task and Sketch of the PRP Model and Its Predictions (A) Scheme of the main PRP effect. The vertical axis labels RT. The column on the left indicates the first task, and each coloured box within the column represents a different stage of processing: P component (dark green), C component (red), and M component (blue). The series of columns on the right indicate the processing time for task 2 at different delays (Δ), labelled on the x-axis. For each column, the three different boxes represent the three different stages of task 2: P component (green), C component (orange), and M component (cyan). As Δ progresses, the P component starts later. All components can be performed in parallel except for the C component, which establishes a bottleneck. This results in the following predictions: (1) response to the first task is independent of Δ, and (2) the RT2 (from onset of the trial) represented by the black line, is unchanged for small Δ while at sufficiently large Δ (noninterference regime) it increases linearly, with a slope of one, with Δ. (B) The predicted RT1 and RT2 (from trial onset) as a function of Δ is represented by the grey and black lines, respectively. (C) The model also establishes definite predictions for experiments in which one of the tasks is changed. The six different panels indicate all possible manipulations: first task changed (left column) or second task changed (right column) and whether the change affects the P component (first row), C component (middle row), or M component (bottom row). The changed component is labelled with a highlighted box and with an arrow. For simplicity, we assumed that the task manipulation always increases the duration of one component. RTs before the manipulation (which are the same across all panels) are represented with a solid line, grey for RT1 and black for RT2, and the RTs of the manipulated task are labelled with a dotted line with the same colour code. If the first task is changed (left column), different effects are observed depending on whether the change is in the M component or in the P–C components (which cannot be distinguished with this manipulation). If the M component is affected (bottom row), RT1 changes, but the response to the second task is unchanged. If the locus of the change is in either the P or the C component (middle and top rows), there is a larger delay until execution of task 2 and the following effect is observed: for small Δ (interference regime), RTs are increased and the regime of interference is increased, which is indicated by a shift of the kink to the right. If the second task is changed (right column), different effects are observed depending on whether the change is in the P component or in either the C or M component. If the change is in the P component (top row), for small Δ there is no net change in the response to the second task (because there was a wait at the end of the P component so extending it does not change total time execution), but there is less wait and thus the kink is shifted to the left. If the change is made in either the C or M component (middle and bottom rows) the result is a rigid shift, which is independent of Δ. By performing experiments in which the two tasks are presented in different orders, all task components can be differentiated. All task manipulations, according to the PRP model, should fall into one of the three categories, perceptual, central, or motor, each defined by its characteristic RT signature.

In addition to tests of additivity, a useful experimental technique to address the separable nature of different components and to understand their organization in time, is the interference analysis, in which the task of study (the number-comparison task) is performed together with a probe task (the tone task). The delay in the onset between the two tasks is controlled experimentally, and to achieve a full separation of the three components, the two tasks must be presented in both possible orders (Figure 3). Under the assumptions of the PRP model, the P and M components can be carried out in parallel with another task, but the central stage is the only one that provides a bottleneck, in the sense that the central component of each task cannot be carried out simultaneously. From these premises, one can predict the curve giving RTs for the first and second tasks (RT1 and RT2, respectively) as a function of delay, and how it changes with a manipulation of the P, M, and C components of either task. The sets of predictions and a sketch of the logic are presented in Figure 3. The aim here is thus to associate the experimental manipulations (notation, distance, and response complexity) to the different components in the PRP model (P, C, and M) by analysing the changes in mean RT with delay. Here and throughout the paper, we follow the convention that RTs to both tasks are reported from trial onset.

We begin by describing the mean RT results when the number task (for which experimental parameters were varied) was performed first, and the tone task came second. The PRP model predicts that each of the manipulated variables of notation, distance, and response complexity should have a main effect on the first number-comparison task, but only some of those effects (those that affect P and C components of the first task) should propagate to the RT2, and should do so only at short interstimulus delays (Δ) (Figure 3).

To evaluate these predictions, mean RTs were calculated within each condition and each subject, and submitted to ANOVAs with subjects as a random factor and delay and the variable of interest as within-subject factors. The detailed results of those ANOVAs are reported in Table 4. In the text, we merely draw attention to the main points.

**Table 4:**
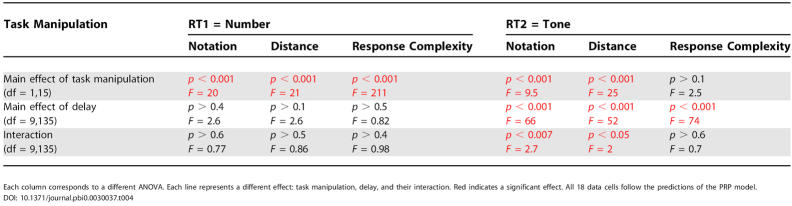
Results of the ANOVAs of the Interference Experiments: Number Task Followed by Tone Task

Each column corresponds to a different ANOVA. Each line represents a different effect: task manipulation, delay, and their interaction. Red indicates a significant effect. All 18 data cells follow the predictions of the PRP model

DOI: 10.1371/journal.pbi0.0030037.t004

The ANOVAs on number-comparison RTs (RT1) revealed the expected main effects of number notation (74 ms, slower for verbal than for Arabic numbers), numerical distance (91 ms, slower for close digits than for far digits), and response complexity (175 ms, slower for two-tap responses than for one-tap responses). There was no main effect of Δ, and none of the task effects interacted with delay. These results suggest that, as requested, participants performed the number comparison as task 1 independently of the delay of presentation of the subsequent tone task.

**Figure 4:**
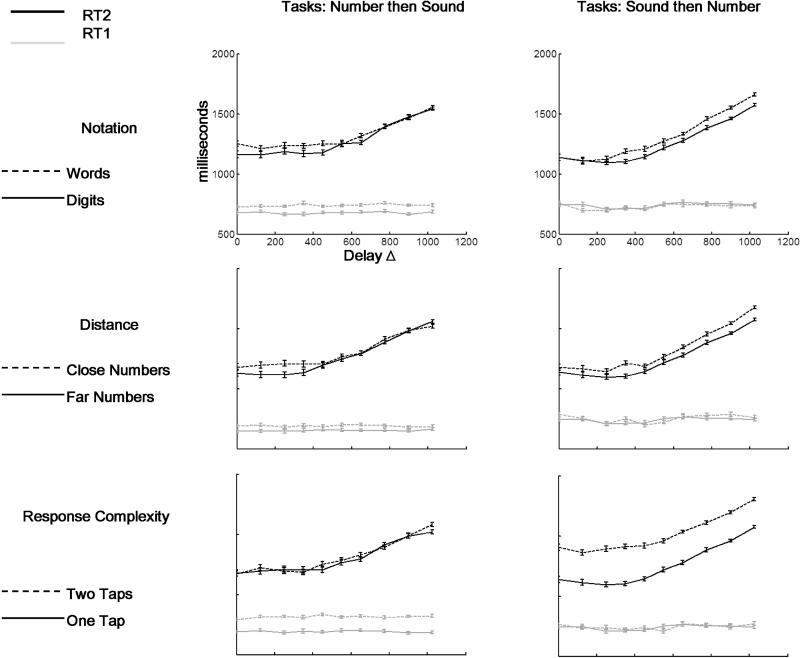
Dissociating P, C, and M Components by Their Interference Patterns In the left column the number task is performed first and the tone task second. In the right column the tone task is performed first and the number task second. In both cases, the number task is manipulated by the three factors of notation, distance, and response complexity. In all panels the code is identical: RT1is coloured grey while RT2 is coloured black. The “easy” condition is represented by a solid line and the “difficult” condition by a dotted line. All the data can be explained in terms of the PRP model: notation (top row) affects the P component, distance (middle row) affects C, and response complexity (bottom row) affects M (see also Tables 4–6 for statistics, and note the agreement with the predicted RTs shown in Figure 3).

Similar ANOVAs on tone-decision RTs (RT2) revealed a main effect of delay, characteristic of the PRP phenomenon. As shown in Figure 4 (left column, black solid curves), RTs were independent of delay up to a certain value, then began to increase linearly with further increases in delay.

**Table 6:**
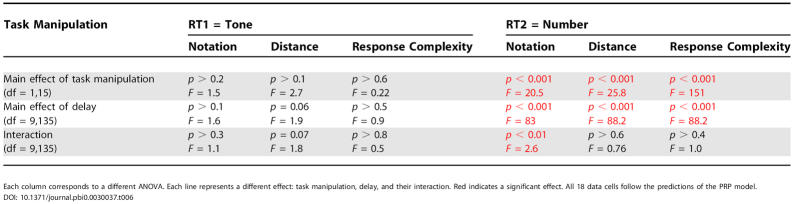
Results of the ANOVAs of the Interference Experiments: Tone Task Followed by Number Task

Each column corresponds to a different ANOVA. Each line represents a different effect: task manipulation, delay, and their interaction. Red indicates a significant effect. All 18 data cells follow the predictions of the PRP model

DOI: 10.1371/journal.pbi0.0030037.t006

Crucially, our three experimental factors had differential effects on those two segments of the RT curve. Notation and distance showed both a main effect and an interaction with delay (Table 4).

As a further test, we analysed the data for short delays, within the interference regime (Δ ≤ 350 ms) and long delays (Δ ≥ 600 ms) (Table 5). For the notation and distance manipulation, when collapsing the data across all short delays, there was a significant effect of both factors (respectively 87 ms and 100 ms). For long delays RTs were no longer affected by those variables. These features are characteristic of effects that affect either the P or the C components of a task (see Figure 3).

**Table 5:**
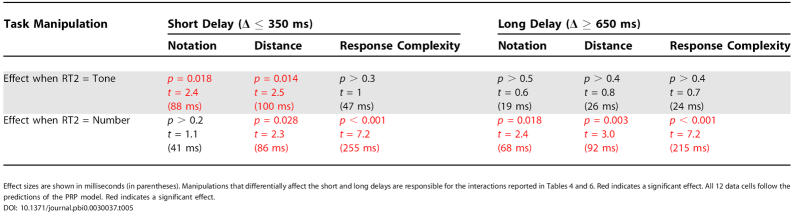
*t*-Tests to Study the Effect of Each Manipulation on RT2 within the Regime of Interference (Short Delays) and within the Regime When the Two Tasks Are Performed Independently (Long Delays)

Effect sizes are shown in milliseconds (in parentheses). Manipulations that differentially affect the short and long delays are responsible for the interactions reported in Tables 4 and 6. Red indicates a significant effect. All 12 data cells follow the predictions of the PRP model. Red indicates a significant effect

DOI: 10.1371/journal.pbi0.0030037.t005

The situation was quite different for the response-complexity variable. The ANOVAs did not reveal either a main effect of response complexity or an interaction with delay on the RT2 (see Figure 4, bottom left, black curves; see also Table 4). Thus, none of the larger (175 ms) effect that was observed on the first task was propagated to the second task. This result is confirmed by the *t*-tests, where we did not observe a significant difference either in the short delays or in the long delays (see Table 5). This is characteristic of a variable that affects the motor stage of processing.

We now describe the mean RT results when the tone task was performed first, and the number task (for which experimental parameters were varied) came second. In this case the PRP model predicts that there should be no effect of the manipulated variables of notation, distance, and response complexity on the first tone task; in addition, the RT2 should exhibit a constant increase (independent of delay) when the change affects the M and C components and should change only for large delays when the change affects the P component (see Figure 3). As described above, to evaluate those predictions, mean RTs were calculated within each condition and each subject, and submitted to ANOVAs with subjects as a random factor and delay and the variable of interest as within-subject factors (see Table 6).

The ANOVAs on the tone task RTs (first task, RT1) revealed no effects on the task manipulation, as predicted by the PRP model because response to task 1 should be independent of the nature of task 2. The ANOVAs on the number-comparison RTs (second task, RT2) again revealed a very significant nonlinear effect of delay characteristic of the PRP effect. In addition, for the distance and response-complexity manipulations, we observed a task effect that did not interact with delay (see Table 6), typical of central and motor manipulations. For the notation manipulation, we observed a task effect that interacted with delay, typical of perceptual manipulations.

These observations were consistent with the *t*-tests performed for short and long delays. When data were collapsed across all short delays for the distance and motor manipulation, there was a significant effect of both factors (respectively 86 ms and 255 ms). In contrast, there was no significant difference in RT for the number task for the different notations for small delays (see Table 5). For all comparisons there was a significant effect for long delays: notation (68 ms), distance (92 ms), and response complexity (215 ms). Thus, the notation effect behaves with the characteristics of a variable that affects the P component, and combining this analysis with the prior in which the number task came first, we observe that each manipulated variable affects a different component: notation affects P, distance affects C, and response complexity affects M (compare the predictions of each stage, Figure 3; and the data resulting from each manipulation, Figure 4).

The dependence of RT on delay follows the prediction of the PRP model for all conditions, task manipulations, and task orders. However, we find a small departure from the model when we compare the mean RTs for both tasks when they were presented either first or second at the maximum delay (1,025 ms). In both cases we find that the response is slower when the task is presented first: number task, 756 ms when presented first and 678 ms when presented second; tone task, 720 ms when presented first and 518 ms when presented second. Thus there is a fixed component (independent of delay) of approximately 150 ms, which needs to be added to RT1 to fully explain the data.

### Detailed Analysis of the Distribution of RTs

#### Effect of the different manipulations of the number task

**Figure 5:**
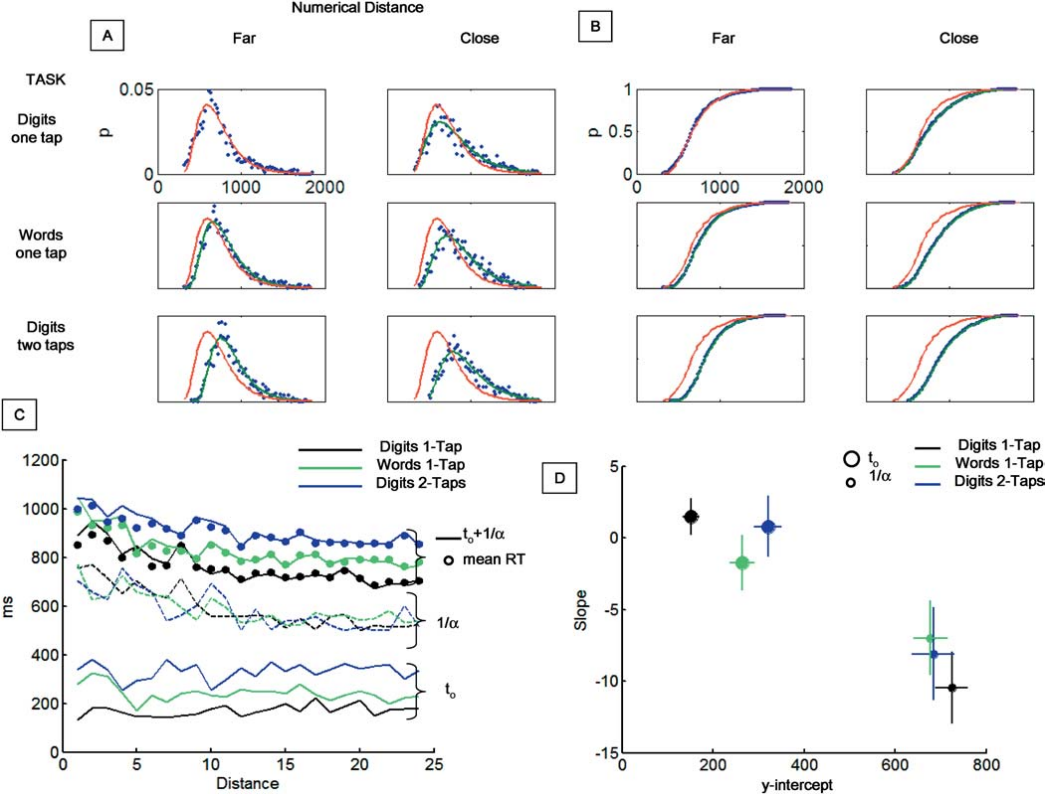
Dissociating Parallel and Serial Components by RT Distributions (A) RT histograms (when the number task was presented first) fitted by a simple random-walk model, separately for far distances (left column) and close distances (right column) and for the three different tasks: Digits 1 Tap (top row), Words 1 Tap (middle row), and Digits 2 Taps (bottom row). (B) Cumulative plots of the same data. The effect of both notation and response appears to be a shift of the distribution to the right while the distance effect is a change in the slope. Within each panel, we have overlapped the corresponding fit (blue line) and the fit to the easiest condition—Digits 1 Tap, Far Digits (red line)—to make the change between the different distributions apparent. (C) The two fitted values (fixed delay and integration time) as a function of numerical distance for the three different tasks. The integration time decreases with distance, but it is independent of the tasks. In contrast, the fixed delay does not change with distance but changes with the task. The summed delay plus integration time fit the mean reaction times for each distance (solid circles). (D) Statistics performed on the fit reveal that the fixed delay has a slope not significantly different from zero (i.e., it does not depend on distance), but it changes with task. In contrast, the integration time is significantly different from zero, but it does not change with task.

The shape of the RT distributions (for correct trials) was analysed for each task when it was presented first. For the number task we analysed six different cases corresponding to the three different manipulations (Digits 1 Tap, Words 1 Tap, and Digits 2 Taps), and two levels of numerical distance: close distances (≤12) and far distances (>12). For each of these distributions, the histograms of RTs and their cumulative distributions were calculated, and the latter were fitted to a simple model of RTs. The model was based on a fixed onset delay, *t*_o_, followed by a forced random walk to a threshold *T* with slope α and diffusion constant σ (Figure 5). The fixed delay (*t*_o_) corresponds to the sum of the P and the M components (see Figure 1).

The applicability of random-walk models to RT data has been widely studied in numerous tasks [18,19,20,21,22,23], including the number-comparison task [17]. While there are a large number of variants (see Discussion), allowing us to capture further details of the data at the expense of increases in theoretical complexity, our approach here is to remain with a model as simple as possible, whose sole purpose is to separate stochastic and invariant contributions to reaction times. The parameters were determined as follows. *T* can be set to one without loss of generality. For simplicity, we assumed that σ was the same for all six experimental conditions, while α and *t*_o_ could vary (we verified that none of the results depended qualitatively on the particular choice of σ). The best-fitting values were determined by exhaustive search using a minimum-squares criterion. The value of 1/α characterizes the integration time (which explains all the variance), while *t*_o_ captures fixed components that do not contribute to the variance. Thus, our purpose was to test the prediction of our model that the notation and response-complexity manipulations should affect the parameter *t*_o_ while the distance manipulation should affect the parameter α.

Figure 5 shows the fitted distributions of RTs corresponding to the three different tasks: Digits 1 Tap (Figure 5A and 5B, first row), Words 1 Tap (Figure 5A and 5B, second row), and Digits 2 Taps (Figure 5A and 5B, third row). For each of these tasks, we have separated the data corresponding to the close distances (right column) and the far distances (left column). The fit was accurate, with the exception that it was smoother than the real data and thus did not fully capture a fairly abrupt peak at the modal response. The shapes of the distributions appeared to change in two qualitatively different manners. For fixed distances (same column) but changing task, the distributions shifted in time. Conversely, for fixed task (same line) but changing the numerical distance, the distribution became wider.

For a finer-grained analysis, and to test the significance of this phenomenon, we binned the data in 24 different bins based on their distance to the reference 45 used for numerical comparison. For each bin, we calculated the α and *t*_o_ that provided the best fit. We found that the *t*_o_ changes from task to task but does not depend on distance. In contrast, 1/α does not change across tasks but changes with numerical distance (Figure 5C). To test this, we performed a linear regression of both parameters as a function of distance, thus producing two estimates (the slope and the *y*-intercept at *x* = 0) (Figure 5D). For *t*_o_ the value of the slope (for the three tasks) does not differ significantly from zero (*p* > 0.3) and the value of the intercept differs significantly across tasks (*p* < 0.001). In contrast, for 1/α the intercept is not significantly different across tasks (*p* > 0.5) while the slope is significantly different from zero (*p* < 0.001) Thus, response complexity and notation manipulation affect *t*_o_, while numerical distance affects 1/α. These results are consistent with the prior analysis, which showed that response complexity and notation manipulations did not significantly affect the interquartile range (another measure of dispersion) while the distance manipulation did significantly change the interquartile range.

#### Prediction of the distribution of RT2s

Here we try to explain the precise shape of RT2s, by combining, based on the PRP model, the distributions obtained for each task when presented first. If the two tasks were completely sequential, then the resulting distribution would be simply the convolution of the two original distributions. However, the PRP model states that only the C component is sequential, and, thus, because some operations can be done in parallel, the resulting RT2s are shorter than expected from a convolution. The operation performed is not completely trivial and is described step by step in Materials and Methods. The only essential point is that this calculation cannot be performed by simply knowing the RTs to each task, but also requires an estimate of the duration of the M component of the first task (M1) and the P component of the second task (P2). These durations are not directly accessible to measurement, but they can be estimated as a result of the fitting of the distribution of RT2. Thus, confronting the distributions of the first and second tasks provides access to the otherwise hidden durations of the postulated component stages, allowing further tests of our model.

**Figure 6:**
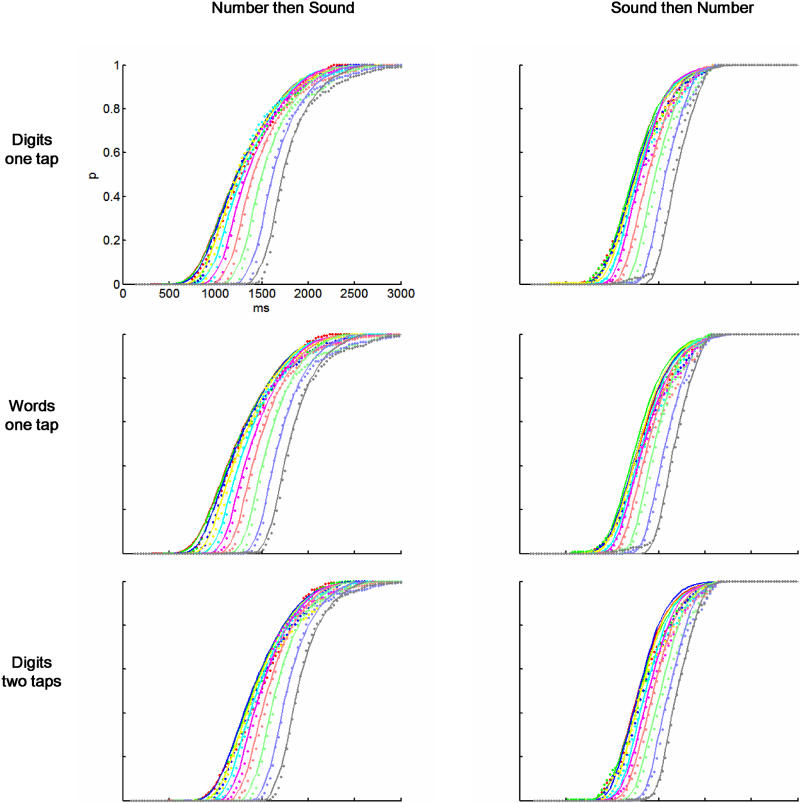
Predicting the Distribution of RTs to the Second Task from the PRP Model Left: Cumulative plots of RTs to the number task when it is presented second (dots) and the predicted distribution based on the PRP model (solid lines). Each curve (coded in different colours) represents one of the ten possible values of Δ. Right: Same data for RTs to the tone task when it is presented second (dots) and the predicted distribution from the PRP model (solid lines). Each row corresponds to a different task: Digits 1 Tap (first row), Digits 2 Taps (second row), and Words 1 Tap (third row). Each panel was fit with three parameters: M1, P2, and a fixed delay.

For each task (Digits 1 Tap, Words 1 Tap, and Digits 2 Taps) we tried to fit the 20 distributions of RT2 (ten for each value of the delay and the two possible orders of the tasks (tone–number or number–tone) from the distributions of RT1, with *P2* and *M1* as free parameters. We found that with these parameters alone, the data could not be fitted (there were no values of the parameters that gave mean square residuals less than 0.3 for all distributions, and the fitted curves were not similar at all to the real data). It seemed evident that the problem was that the predicted distributions were shifted in time with respect to the original distributions, and thus we decided to add one parameter, *T*_d_, a rigid shift in time of all distributions of RT2 (see Discussion for the rationale of this parameter). We then found good fits for the ensemble of distributions (Figure 6, mean square residuals < 0.015) with the following values of *T*_d_: Digits 1 Tap, 125 ms; Words 1 Tap, 125 ms; and Digits 2 Taps, 75 ms.

**Figure 7:**
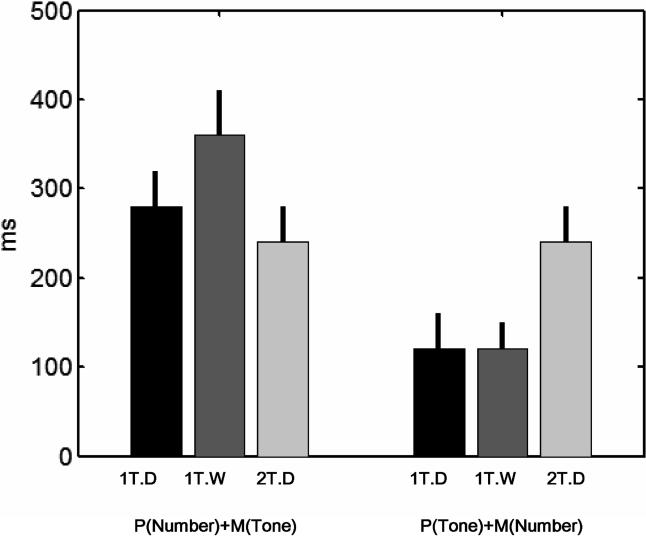
Parameters Obtained from the PRP Fitting and Their Task Dependence The PRP fitting allowed us to estimate the values of P2 + M1. Depending on which task is presented first, we can calculate *P(Number)* + *M(Tone)* (left bars) or *P(Tone)* + *M(Number)* (right bars). *P(Number)* + *M(Tone)* changes with notation manipulation but not with response manipulation. Conversely, *P(Tone)* + *M(Number)* changes with response manipulation but not with the notation manipulation. Furthermore, the left bars are consistently higher than the right bars, suggesting that visual perception of digits and words takes approximately 150–220 ms longer than auditory perception of a single tone.

Each fit provides the parameters *P2* and *M1.* When the number task was second, the parameters *are P(Number) and M(Tone).* When the tone task was the second, the fit parameters are *P(Tone)* and *M(Number).* The obtained values of the square residuals for different parameters were not sufficient to actually calculate precisely each parameter, since the fit was unstable in the *P2 − M1* direction (i.e., it did not change much if both parameters were changed but their sum was kept constant), but they were sufficient to calculate their sum (Figure 7). In agreement with our previous observation, we found that the notation manipulation affects *P(Number)* + *M(Tone)* (Figure 7, left) but not *P(Tone)* + *M(Number)* (Figure 7, right). In contrast, and also consistent with our previous findings, the response-complexity manipulation affects *P(Tone) + M(Number)* (Figure 7, left) but not *P(Number) + M(Tone)* (Figure 7, right).

Finally, the parameters obtained from the interference experiment may be compared to those of the previous fit, which was based on the shape of the distributions of RTs for the first task, and which yielded estimates of 1/α (the time of integration) and *t*_o_ (a fixed delay). As expected from our model, across the different conditions summarized in Table 7, we observe that *t*_o_ is always approximately equal to the sum of the durations of the P and M components, while 1/α is equal to the duration of the C component. This provides further evidence that the process of accumulation of evidence does indeed constitute the characteristic bottleneck (the C component) in dual-task experiments.

**Table 7:**
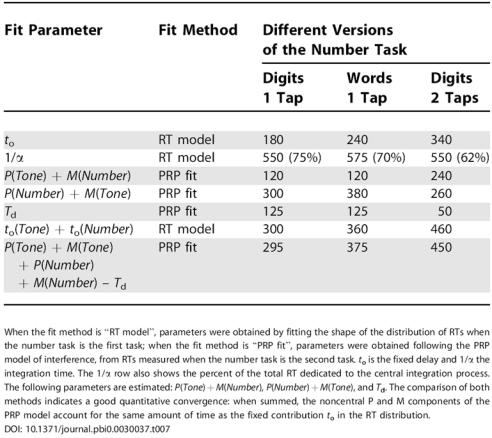
Fitted Parameters

When the fit method is “RT model”, parameters were obtained by fitting the shape of the distribution of RTs when the number task is the first task; when the fit method is “PRP fit”, parameters were obtained following the PRP model of interference, from RTs measured when the number task is the second task. *t*_o_ is the fixed delay and 1/α the integration time. The 1/α row also shows the percent of the total RT dedicated to the central integration process. The following parameters are estimated: *P*(*Tone*) + *M*(*Number*), *P*(*Number*) + *M*(*Tone*), and *T*_d_. The comparison of both methods indicates a good quantitative convergence: when summed, the noncentral P and M components of the PRP model account for the same amount of time as the fixed contribution *t*_o_ in the RT distribution

DOI: 10.1371/journal.pbi0.0030037.t007

## Discussion

We proposed a basic model that relates the organization of parallel and serial components and the process of accumulation of evidence to reach a decision. The model, although simple, results in a wide number of predictions that, as we have shown, hold over a vast variety of manipulations. We show that the perceptual transformation of sensory information into an abstract quantity representation can be carried out in parallel with another task and is a low-variability process (whose variability does not increase with the mean); that the accumulation of evidence establishes a bottleneck and is an intrinsically variable process; and that the execution of the response constitutes yet another parallel, low-variability process. Our data suggest that the integration of evidence in time to reach a decision constitutes the only central process in a simple cognitive task that links perceptions to actions.

### Validity of the PRP Model

While dual-task experiments (in which two tasks are presented at variable delays) allow different interpretations, experiments in which one of the two tasks is parametrically manipulated provide a severe test of the PRP model [12,13,16]. Indeed, the simple hypothesis that the central module is the only serial stage results in concrete predictions about the dependence of mean RTs on delay [16]. In different cognitive tasks, the PRP model was successfully used previously to identify and dissect different processing components. For example, in a detection experiment where the brightness and the probability of target occurrence were manipulated, it was shown that brightness behaved as a perceptual component while frequency showed the characteristics of a C component [31]. PRP models have also been used to show that word selection involves C components while phoneme selection behaves as an M component [32,33].

Here we have tested, within the number-comparison task, three different manipulations, in the two possible orderings of the sequential tasks, thus providing an exhaustive test of the model. Our finding that all manipulations fall reliably within one of the PRP components provides strong evidence of the generality of this phenomenon. In addition, while it had been shown previously that the distribution of dual-task RTs was wider than that predicted by noninterfering processing of the two tasks [34], precisely adjusting this distribution based on the PRP model, to our knowledge, has not been done before. Our analysis of the distribution of RTs for the second task based on the distributions for each individual task when presented first implies that the model can explain not only the mean RTs, but also their entire distribution. Since the model is parametric, fitting it to the data yields absolute measurements of the duration of the central stage and of the sum of perceptual and motor stages (Table 7). Those measurements, which we obtained consistently by two different means (analysis of single-task RT distributions and of the PRP interference pattern), are consistent with previous experiments using the additive-factors method [29].

A striking result, however, is the duration of the C component, which even in a simple task represents about 70% of the total RT. Considering the simplest version of our task (comparison of Arabic numerals, one tap), our results indicate that 180 ms is taken by the sum of P and M components, while a full 550 ms is taken by the C component. Previous event-related potential experiments suggested that it takes approximately 190 ms to identify an Arabic digit and begin to access a quantity representation [29]. The present evidence indicates that this notation-dependent stage is absorbed during the PRP delay and thus belongs to the P component. Altogether, the evidence suggests that the central stage starts after digit identification and goes on all the way to the actual key press.

While all the PRP predictions held, the only discrepancy with the model arose from an unexpected slowness of responses to the first task. As predicted, RT1 was independent of the delay. However the mean RT was larger than found previously when subjects performed only the number-comparison task [35]. Even within our experiment, it was larger than the time taken to perform the same task when it was presented second at a delay of 1 s (in the noninterference regime). This discrepancy also became evident in the convolution of the two distributions, where the fitting turned out to be impossible without a translation in time, but became very accurate once this translation was added to the fit. Previous PRP experiments have also observed a similar slowing down of the first task, independent of Δ [36]. Thus we believe that a correction needs to be made to the PRP model. There are at least two possible and not exclusive rationales for this correction. First, temporal attention could be involved. The presentation of the first task could act as a primer in time for the second task. Indeed, it has been shown that reaction times decrease when subjects know the precise timing of stimulus occurrence [37]. Second, executive attention might also have to be engaged before performing the first task, in order to prepare for the instructions of performing the two tasks in a specific order and with specific responses. Thus, two components, a structural central bottleneck and a central task-setting component, may contribute to the delay in the dual-task paradigm [14,38].

Here, as in other PRP experiments, we have designed the tasks in order to maximally separate the inputs and outputs to the system (different perceptual modalities and different response hands). Under these conditions, as described above, we still find a source of central interference. Moreover we find that the transformation from a word form to an abstract semantic representation does not participate in this central process, nor does the execution of two consecutive and repetitive motor actions. The generality of these findings, however, has obvious bounds. We do not state here that any motor manipulation should result in a change in a parallel component; more complex motor responses, however, might require central supervision and create a bottleneck. Similarly, while we claim that mapping a word form to an abstract number representation can be done in parallel, we do not mean that it would not interfere with any possible stimulus. Finally, under some situations that lead to high automaticity, either through extensive training [39,40] or very consistent stimulus–response mapping, the central bottleneck may be negligible [41,42,43].

### Alternative RT Models

There is a vast literature on the analysis of the shape of RT distributions as a source of knowledge about the human information-processing system, and many different models of these distributions have been proposed [20,21,23,26,44,45,46,47,48,49,50,51]. Here we did not intend to fully test the validity of the different models or to see which provided a better explanation of our data. We rather chose a simple model that contains the essence of a stochastic integrator and tried to use it to understand the effects of different manipulations. Our most important finding is that the distance manipulation, which is the only one to show interference, as revealed by the PRP experiment, is also the only one to change the stochastic integration time. Conversely, the manipulations that show no interference only affect the fixed delay. Thus there is a consistent parsing of the task from both methods. There are, however, several variations in the model that would be particularly interesting to test in this condition. First, as alternatives to the random-walk model with fixed mean and variance that we adopted here, one may propose a noise-free integration whose slope varies from trial to trial [52] or a diffusion model with variance in the drift [45]. Distinguishing these models may provide a way to measure the timing of the flow of information between perceptual stages and central stages. Do sensory systems provide only one vote to a noisy decision machinery, or do they rather provide a series of stochastic votes, which the decision machinery accumulates? And if the latter, what is the sampling time of communication between both systems?

A second important type of alternative to our model concerns the nature of the central process. Instead of a unique integrator, there might be a network of interacting integrators with lateral connections, which collectively implement the decision-making process and whose interactions create a functional bottleneck [23]. The existence of rare but attested cases in which two response-time tasks can be performed in parallel without cost [41,42] might seem to favour the existence of multiple integrators, but it is also possible that highly trained sensorimotor tasks can eventually be triggered directly, without going through an accumulation-based decision stage [53,54].

Finally, for simplicity our model assumed a constant decision threshold *T.* In a more complicated model, the value of the threshold might be changeable. Such a feature might be needed to fit the results of experiments in which one varies the prior probability of a given response or its associated reward (variables that were fixed throughout our experiment). For example, in a go/no-go experiment involving digit comparison, in which the probability of a response was fixed at a controlled probability p_go_, it has been shown that p_go_ affects *T,* but not the drift rate (α) [17]. Even in experiments with fixed response probabilities, subjects might continually adjust their threshold, lowering it after a successful response and increasing it after an error [55]. Such adjustments might capture another characteristic feature of RTs, which is their intrinsic autocorrelation structure and, in particular, their increase following errors [26,56].

While typically even simple tasks result in highly variable distributions of RTs, under some particular circumstances, including extensive practice, very precise (almost invariant) distributions of RTs can be obtained, e.g., in subjects trained to estimate a fixed duration [57]. It has also been shown that task modifications can lead to fixed delays, i.e., increases in RT that do not change the variance [57]. These findings support the idea that certain mental processes, as we propose here, can be carried out with negligible variance. They imply that variability in RT does not result merely from an intrinsically noisy neuronal machinery but rather from the computation underlying each process. Here, based on our results, we propose a hypothesis that needs further testing: that the central processes that involve integration of information represent the bulk of the variance while perceptual and motor processes are highly reliable. In particular, we predict that if a task can be performed in an invariable fashion it should also be automatic, in the sense of becoming immune to central interference.

### Cerebral Substrates of the Different Components

While we characterized the different processing stages through behavioural observations, it is an essential issue to relate these findings to brain anatomy and physiology. At the single-task level, the neurophysiological bases of simple perceptual decision making have been widely studied in tactile- [58,59,60] and visual-discrimination tasks [53,54,61,62,63,64,65,66]. These studies have revealed direct physiological correlates of the accumulation process postulated in formal RT models. Some neurons appear to code for the current perceptual state. For instance, neurons in the middle temporal area (area MT) appear to encode the amount of evidence for motion in a certain direction [25,63]. Other neurons, distributed in multiple areas including posterior parietal, dorsolateral prefrontal, and frontal eye fields, appear to integrate this sensory information and thus show stochastically increasing firing rates in the course of decision making [25,61,67]. In agreement with the accumulation model of decision making, the rate of increase varies with the quality of sensory evidence [25,61,68], and the response is emitted when the firing exceeds a threshold [66]. Furthermore, accumulation of information about the upcoming response appears in the firing train after a latency of about 200 ms [69,70], which is relatively fixed for a given task and might thus index the duration of the initial perceptual stage.

In humans, a similar indicator of accumulated evidence towards a motor decision is provided by scalp recordings of the lateralized readiness potential (LRP) [71]. The LRP is calculated as the difference in event-related potentials between electrodes overriding left and right motor cortices. In a bimanual task, this index shows a monotonously increasing deviation predictive of the side of the upcoming motor response, and whose intensity reflects the accumulated amount of evidence [72,73]. In numerical comparison, the LRP starts approximately at 200 ms [74], again compatible with a fixed perceptual delay. While the LRP component is localized to motor and premotor cortices, another event-related potential component more broadly distributed across the scalp, the P3 is also associated to postperceptual processes [71,72] and shows a continuous, accumulation-like increase as a function of numerical distance in a comparison task [75]. Thus, both LRP and P3 might reflect the accumulation of evidence observed in monkey electrophysiological studies in distributed parietal and frontal regions.

Indeed, functional magnetic resonance imaging studies of the comparison task show that intraparietal and precentral cortices are systematically activated and that their activation correlates with the distance between the objects to be compared [76]. This bilateral parietal and frontal system has been identified as a shared response selection system across a diversity of input modalities and across different types of stimulus–response mappings [77]. There is a debate, however, concerning the universality of this system, because at least some studies have found variable sites of activation associated with response selection in different tasks [78]. For instance, in an auditory paradigm dissociating the amount of sensory evidence and the response accumulation process, the former was associated with superior temporal cortex and the latter with anterior insula and opercular frontal cortex [79].

What happens to those physiological decision processes during dual-task performance? At present, we know of no neurophysiological study and only a handful of human physiological studies of the PRP phenomenon. In event-related potentials, when C and P components were manipulated, perceptual manipulation led to a change in the P2 component (generally associated with perceptual processing), while the central manipulation affected the amplitude and the onset of the P3 component [31]. The LRP is also delayed during the PRP, in tight correlation with RT [80]. Finally, functional magnetic resonance imaging, a time-insensitive measure, showed that in the interference regime of the PRP there is no increase in activation relative to performing the two tasks independently, even when searching at a low threshold within regions of interest, which included the prefrontal cortex, the anterior cingulate, and supplementary motor area [36]. This result suggests that the PRP does not result from active executive monitoring processes, but rather from a passive queuing of the second task, as proposed in the present model.

Altogether, neurophysiological and brain-imaging studies suggest that, beyond an initial perceptual delay of about 200 ms, there begins a process of accumulation of evidence, which involves the joint activation of a distributed network of areas, with partially changing topography as a function of the nature of the task, but with frequent coactivation of parietal and premotor regions. Our results suggest that this accumulation system is responsible for establishing the PRP bottleneck. This bottleneck might occur because the cerebral accumulation system is broadly distributed and largely shared across tasks, and thus must be entirely “mobilized”, at any given moment, by whichever task is currently performed (for a simulation of this process, see [81]). This neuronal implementation of our model leads to a precise electrophysiological prediction, which could be tested in further research: the accumulation neurons in the lateral intraparietal area and frontal eye field, in an animal trained to perform a pair of PRP tasks, should show two successive stages of accumulation staggered in time; in humans, this might be reflected in a rigid, nonoverlapping sequence of two LRP or P3 event-related components, whose respective durations should covary with the RTs to the two tasks.

## Materials and Methods

### 

#### Participants

A total of 42 participants, all right-handed, were involved in this study (24 males). Sixteen participants (aged 25 y ± 5 y) performed the experiment in which the tone task was presented first, and the other 16 (aged 24 y ± 4 y) performed the experiment in which the number-comparison task was presented first. Ten participants (aged 22 y ± 2 y) performed the numeric task with the addition of the Words 2 Taps condition. Participants were all native French speakers and were remunerated for their participation.

#### Procedure

Participants were asked to perform two tasks, with the clear instruction that they had to respond accurately and as fast as possible to each of them. The delay in the onset of the two tasks changed randomly from trial to trial from 0 ms (simultaneous presentation) to 1,025 ms. Subjects responded to both tasks with key presses, with the right hand for the number-comparison task and with the left hand for the tone task. In the number-comparison task, a number was flashed in the centre of the screen for 150 ms, and subjects had to respond whether the number was larger or smaller than 45. The presented number ranged between 21 and 69, excluding 45. In different blocks, subjects performed three different versions of the number task. In the first version, the number was presented in Arabic digits and subjects were asked to respond by tapping once over the corresponding key (Digits 1 Tap). In the second version, the number was presented as a written word (in French), and subjects were also asked to respond with a single key press (Words 1 Tap; we refer to this as the “notation manipulation”). Finally, in the third version, the number was presented in Arabic digits, but subjects were asked to respond by tapping the corresponding key twice (Digits 2 Taps; we refer to this as the “response-complexity manipulation”). Within each block, both the numerical distance between the target and 45 and the delay between the presentation of the two stimuli varied randomly, and trials were presented with an intertrial interval that fluctuated between 2,600 and 3,000 ms.

In each block, which lasted almost 2 min, subjects performed 40 trials. Before the beginning of each block, subjects saw instructions on the screen, which instructed them what the number task would be for this corresponding block. Subjects practiced one block of each task to get familiar with the task. After this brief training, they performed a total of 18 blocks (six for each version) in an approximately 45-min session.

#### Stimuli

Stimuli were shown on a black-and-white display on a 17-in. monitor with a refresh rate of 60 Hz. Subjects sat 1 m from the screen. Stimuli were always presented in the fovea, and their size was 1° for the Arabic digits and 2.5° for the words. Auditory stimuli were pure tones of 150-ms duration and 440- or 880-Hz frequency. Auditory stimulation was provided through headphones.

#### Data analysis

All the analyses described here were done only on correct responses (which comprised 83% of the trials). Since there were two tasks and each task had two possible responses, chance level for this experiment is at 25%. Errors (17%) included errors in either the first or second task and trials in which subjects failed to respond to either of the tasks, or both. One subject was discarded from the analysis because the data clearly revealed that he had not performed the task as required. His RT1 arrived systematically a few hundred milliseconds after the onset of the second task, indicating that he was waiting for both tasks to be presented in order to respond and not, as indicated, responding to both tasks as fast as possible. For similar reasons, for all analyses, trials in which the RTs to the first task were larger than 1,200 ms (<5% of the trials) were excluded. All the statistics were done using the R software package (http://www.r-project.org/, and in all ANOVAs subjects were treated as a random factor. Throughout the paper, RTs for both tasks are, per convention, measured from trial onset, i.e., the onset of the first stimulus.

#### Distribution analysis

RTs were fitted to a model based on a fixed delay onset (*t*_o_) followed by a forced random walk *dV = α · dt + σ · dz* and response emission as soon as *V* reaches a threshold *b* (see Figure 1). Thus the RT is defined by *T_R_ =* inf[*t* ≥ 0, *V*(*t*) ≥ *b*], where *b* is the threshold. This problem (of the first hitting time to an absorbing barrier of a Brownian motion) has been widely studied and can be solved analytically using the Fokker–Planck equation. The probability of hitting threshold for the first time at time *t* is given by the following equation:



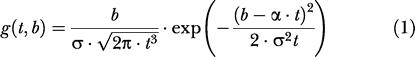



Changing the onset by a fixed delay *t*_o_ and setting the threshold to one simply shifts the distribution, which then becomes



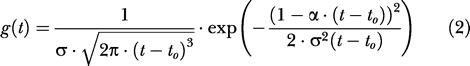



This is the equation we used to fit the RT distributions. All six distributions resulting from the different experimental manipulations corresponding to (Digits 1 Tap, Digits 2 Taps, Words 1 Tap) × (Distance Far, Distance Close) were fit to a fixed value of *σ* and to values of α and *t*_o_, which were allowed to vary across the different experimental conditions. The best parameters were obtained through exhaustive search using a minimum-squares criterion. For each value of *σ*, the best values α and *t*_o_ were found for each experimental condition, and the mean square residuals were averaged across all distributions. It was found that the *σ* that minimized the mean squares deviation across all distributions was 0.018. The changes in the remaining parameters with different experimental conditions, which were of interest to this study, are reported in the Results sections. We repeated this fit for a broad range of *σ* and found that the obtained results did not depend on the choice of *σ*.

#### Predicted distributions based on the PRP model

Here we describe how RTs for task 2 can be predicted based on the distribution of RTs for both tasks when presented first. Because of the presence of the PRP wait (which depends on the value of the response to the first task), this operation is not strictly a convolution. Since the method is not trivial and, to our knowledge, it has not been performed elsewhere, we will describe it step by step:

In a serial sequence of two processes (in which one needs to be finished before the next one starts), each with a probability distribution of RTs given respectively by R_1_ and R_2_, the probability of performing the sequence at time *T* is given by



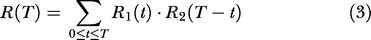



This formula is simply the convolution of the two original distributions.

In a PRP experiment, however, the execution of the two tasks is not serial, since there are both serial (central) and parallel (noncentral) components. The first difference is that task 2 waits not for the complete execution of task 1 but rather for the completion of the P and C components of task 1 (see Figure 3). Hence, the first modification is that the first distribution needs to be shifted by M1 (to account for the real start-up time of the second distribution) *R*_1_^*^(*t*) = *R*_1_(*t* + *M*_1_). The second modification, because of the nature of the PRP experiment, is that task 2 obviously cannot start until it is presented and thus the onset time is actually given by *R*_1_^**^(*t*) = max[*R*_1_^*^(*t*), Δ]**.** Thus the real distribution of onsets of task 2 is given by the accumulated probability of a shifted *R*_1_ up to Δ (which results in a spike at Δ) followed by the tail of *R*_1_^*^(*t*). The spike becomes more pronounced as the delay is larger, and thus the two tasks become independent.

The last consideration has to do with the time it takes to respond to task 2. If Δ is sufficiently large (in the independent regime), the probability of executing task 2 at time *t* is given by *R*_2_(*t*). However, within the interference regime (for small Δ), P2 (or part of it) has been executed by the time that the P and C components of the first task (which corresponds to *t* − M_1_) are finished (see Figure 3). The distribution coincides with *R*_2_ at *t* = Δ, but as *t* increases, part of the P component of task 2 has been carried out and this saturates at *t* = Δ + P2. Thus the probability of executing task 2 at time *t*_2_ given that task 1 has been executed at time *t* + M_1_ is given by *R*_2_(*t*_2_), where *t*_2_ = min[(*t* − Δ), P2] + *t*. This formula is only valid for *t* > Δ, but this is not important because in any case *R*_1_^**^(*t*) **=** 0 for *t* < Δ. The important issue, however, is that this transformation depends on *t* and thus the sum described in equation 3 is not strictly a convolution. We can still define *R*_2_^*^(*t,T*) = *R*_2_[*T* − *t*_2_(*t*)] as the probability of completing task 2 in time *T − t* given that task 1 has been completed in *t* + M1.

The final formula (adapting equation 3 after all the transformations) then becomes



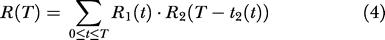



Since all these transformations depend on Δ, M1, and P2, this prediction is parametric. The data were fit by exhaustive search according to mean squares criteria. We fitted all the data (for each task and for all the different values, to obtain the values of M1 and P2). As described in Results, this model was not sufficient to fit the data (note that we are simultaneously fitting a family of 30 curves), so we included a third fixed delay parameter (*T*_d_) to the fit. With the inclusion of the parameter *T*_d_, the errors, measured as the mean square residual (i.e., the mean of the squares of the difference between the data and the fit across all the points of the ten distributions corresponding to all possible delays) were consistently below 0.015 (20 times smaller than could be found without the inclusion of this parameter), and we observed a parabolic type of distribution, with a clear minimum (reported in Figure 7) when plotting the error as a function of P2 + M1. When data were plotted in the orthogonal direction (P2 − M1), however, the fit was unstable with different local minima.

## Abbreviations

Δ: interstimulus delay
C component: central component
LRP: lateralized readiness potential
M component: motor component
M1: motor component of the first task
P component: perceptual component
P2: perceptual component of the second task
PRP: psychological refractory period
RT: response time
RT1: response time for the first task
RT2: response time for the second task
